# The Characterization of Atrial Fibrillation and Prognostic Value of a Modified 4S-AF Scheme: A Report from the REGUEIFA Community Health Area Registry (Galician Intercentric Registry of Atrial Fibrillation)

**DOI:** 10.3390/jcm14051483

**Published:** 2025-02-23

**Authors:** Javier García Seara, Laila González Melchor, María Vázquez Caamaño, Emilio Fernández-Obanza Windcheid, Raquel Marzoa, Miriam Piñeiro Portela, Eva González Babarro, Pilar Cabanas Grandío, Olga Durán Bobín, Óscar Prada Delgado, Juliana Elices Teja, Evaristo Freire, Mario Gutiérrez Feijoo, Javier Muñiz, Francisco Gude, Eduardo Barge Caballero, Carlos González-Juanatey, on behalf of the REGUEIFA Investigators

**Affiliations:** 1 Cardiology Department, University Hospital of Santiago de Compostela, 15706 Santiago de Compostela, Spain; 2 Cellular and Molecular Cardiology Unit, Institute of Biomedical Research of Santiago de Compostela (IDIS-SERGAS), 15706 Santiago de Compostela, Spain; 3 Centro de Investigación Biomédica Cardiovascular en Red (CIBERCV), Institute of Health Carlos III, 28029 Madrid, Spain; eduardo.barge.caballero@sergas.es; 4 Cardiology Department, University Hospital Lucus Augusti, Institute of Biomedical Research of Santiago (IDIS), 27003 Lugo, Spain; laila.gonzalez.melchor@sergas.es (L.G.M.); olga.duran.bobin@sergas.es (O.D.B.); juliana.elices.teja@sergas.es (J.E.T.); carlos.gonzalez.juanatey@sergas.es (C.G.-J.); 5 Cardiology Department, San Rafel Hospital, 15006 La Coruña, Spain; 6 Cardiology Department, Hospital Álvaro Cunqueiro and Institute of Health South of Galicia (IISGS), 36312 Vigo, Spain; emilio.fernandez-obanza-windcheid@sergas.es (E.F.-O.W.); pilar.cabanas.grandio@sergas.es (P.C.G.); 7 Cardiology Department, Arquitecto Marcide Hospital, 15405 Ferrol, Spain; 8 Cardiology Department, Instituto de Investigación Biomédica de A Coruña (INIBIC), University Hospital of A Coruña, 15006 La Coruña, Spain; miriam.pineiro.portela@sergas.es (M.P.P.); oscar.prada.delgado@sergas.es (Ó.P.D.); 9 Cardiology Department, Montecelo University Hospital, 36071 Pontevedra, Spain; eva.gonzalez.babarro@sergas.es; 10 Cardiology Department, Orense University Hospital, 32005 Orense, Spain; evaristo.freire@sergas.es (E.F.); mario.gutierrez.feijoo@sergas.es (M.G.F.); 11 Cardiovascular Research Group, Department of Health Sciences and Institute of Biomedical Research of A Coruña (INIBIC), A Coruña University, Centro de Investigación Biomédica en Red en Enfermedades Cardiovasculares (CIBERCV), 15006 A Coruña, Spain; javier.muniz.garcia@udc.es; 12 Research Methodology Group, Institute of Biomedical Research of Santiago (IDIS), University of Santiago de Compostela (USC), 15706 Santiago de Compostela, Spain; francisco.gude.sampedro@sergas.es

**Keywords:** 4S-AF score, stroke risk, atrial fibrillation, REGUEIFA

## Abstract

**Background:** The REGUEIFA registry aims to assess the contemporary treatment of patients with atrial fibrillation (AF) in the community health area of Galicia. Due to the prognostic relevance of anticoagulation status, we used it to differentiate patients by adding a category to the stroke domain of the 4S-AF score. **Methods:** A Cox proportional hazards analysis was used to identify the prognostic value of the modified 4S-AF score regarding mortality, cardiovascular mortality, and thromboembolic events. For bleeding events, we used a Poisson regression model to account for recurrent events. **Results:** When considering the stroke risk domain as a categorical variable, the risk stratification for all-cause mortality improved by more than 2 times (stroke risk: 2 vs. 1; hazard ratio (HR): 2.17; 95% confidence interval (CI): 1.03–4.55), *p =* 0.04). According to the Poisson regression model, the stroke risk domain was also an independent factor for hemorrhagic events (HR: 2.83; 95% CI 1.69–4.74, *p <* 0.001). For patients with permanent AF, the mortality rate was more than 2 times higher than that of patients with paroxysmal AF or their first episode of AF (HR: 2.53; 95% CI; 1.53–4.18); *p* < 0.001. Anticoagulation therapy was the only independent domain treatment associated with a reduction in all-cause mortality (HR: 0.41; 95% CI 0.19–0.89 *p <* 0.0023). **Conclusions:** The modification of the stroke risk score to reflect anticoagulation status may improve the characterization and stratification of overall mortality risk, as demonstrated in the contemporary AF cohort from the REGUEIFA study. The permanent form of AF was associated with a higher risk of overall mortality and cardiovascular mortality.

## 1. Introduction

The temporal pattern is the most commonly used characteristic for the classification of atrial fibrillation (AF) and allows patients to be categorized according to relatively arbitrary groups: first-diagnosed, paroxysmal, persistent, long-standing persistent, and permanent AF [1,2]. However, this classification lacks clinical accuracy and does not provide information about other important factors, such as stroke risk, symptoms, cardiovascular risk factors, and left atrial (LA) substrates [3,4,5]. A novel structured pathophysiology-based characterization scheme has been proposed for AF and includes four domains regarding AF and patients [6]. This classification provides a comprehensive characterization of AF and has been evaluated for its prognostic value in the EORP-AF (European Observational Research Programme—Atrial Fibrillation) Registry [7]. Nevertheless, in the EORP-AF program, high geographic variation was detected with great heterogeneity in AF treatment, and patients who were treated in a primary care setting were included. Moreover, the patients were recruited before the 2016 ESC guidelines on AF were established [8]. More recently, the ESC EORP-AF III registry recruited patients between 2018 and 2019 and provided insights for contemporary AF management [9].

The REGUEIFA registry was designed to assess the contemporary treatment of patients with AF in the community health area of Galicia and to provide insights about clinical events and mortality in a 2-year follow-up period [10]. Due to the prognostic relevance of anticoagulation status, we used it to differentiate patients by adding a category to the stroke domain of the 4S-AF score. We determined the prognostic value of the slightly modified 4-S domain characterization and the feasibility of clinical use of this scheme in the REGUEIFA population.

## 2. Methods

### 2.1. Study Design

The REGUEIFA registry is a prospective, observational, multicentre registry from eight centers of a community health area (Galicia) in Northwest Spain. A detailed description of the study design has been published previously [10]. In brief, the study included patients dwelling in Galicia with primary or secondary diagnosis of AF who received a cardiology consultation or were hospitalized patients in a cardiology ward between January 2018 and February 2020.

The inclusion criteria were age > 18 years, AF > 30 s diagnosed by electrocardiogram or an external or implantable Holter monitor, and an AF episode within the last year before recruitment. The AF could be a primary or secondary diagnosis. The included patients signed informed consent forms. The exclusion criteria were secondary and reversible AF and participation in an interventional study that examined the frequency of visits and diagnostic tools.

All patients were followed for 2 years, and the collected information included mortality, cardiovascular mortality, cardiovascular hospitalizations, thromboembolic events, bleeding events, quality of life, and changes in rhythm or rate-control strategy. All events were obtained from the electronic clinical history of the patients based on outpatient visits with a cardiologist, primary care visits, and emergency attendance. The Galician Society of Cardiology is the sponsor and promoter of the registry, which followed the guidelines of the Declaration of Helsinki, the European Union Note for Guidance on Good Clinical Practice CPM/ECH/135/95 and Good Pharmacoepidemiological Practice, and local ethics and norms. The registry was approved by the Ethical Clinical Investigation Committee of Galicia (register number 2016/376).

### 2.2. 4S-AF

The 4S-AF scheme for characterizing patients with AF consists of four domains:

Stroke risk (St)—risk of suffering a stroke; symptoms (Sy)—patients’ symptomatology according to the European Heart Rhythm Association (EHRA) classification; severity of AF burden (Sb); substrate (Su)—control of comorbidities and cardiovascular risk factors and evaluation of the presence of echocardiographic signs of LA dilation. The Su domain was evaluated based on the presence of a high body mass index (BMI ≥ 30 kg/m^2^), heart failure, diabetes mellitus, coronary artery disease, peripheral vascular disease, history of thromboembolic events, kidney disease, neoplasia, hypertension, and chronic obstruction pulmonary disease (COPD)/sleep apnea syndrome.

Mild or moderate dilation of the left atrium (LA) was considered when the LA diameter was greater than or equal to 40 mm. The dilation was considered severe when the LA diameter was greater than or equal to 50 mm. In cases where the LA diameter was unavailable, the data were completed with the available LA volume. Mild or moderate LA dilation was considered when the LA volume was greater than 35 mL/m^2^, and severe LA dilation was considered when the LA volume was greater than 48 mL/m^2^.

The decision to initiate anticoagulation was made by the investigators of the REGUEIFA study in accordance with the recommendations of the 2016 European Society of Cardiology guidelines on atrial fibrillation [11]. We made a slight modification to the 4S scheme, which affects the stroke domain in relation to the anticoagulation status of patients with a CHA_2_DS_2_-VASc score ≥ 1 for men and ≥ 2 for women. We assigned a score of 1 if they were anticoagulated and 2 if they were not anticoagulated. This modification was made because although the CHA_2_DS_2_-VASc score correlates with the risk of stroke and overall mortality [12], both are significantly influenced by the presence or absence of concurrent anticoagulation at different stages of the score. Hence, it seems reasonable to integrate the anticoagulation status into the stroke risk in the 4S scheme. For Cox proportional hazard analysis, the Su domain was recategorized such that a score of 1 corresponds to patients with 1 or 2 points, while a score of 2 corresponds to patients with 3 or 4 points.

### 2.3. Effect of Treatment and 4S

The effect of treatment was evaluated in the following subgroups based on the domains of 4S-AF:

- Patients with stroke risk (St) ≥ 1: Treatment was based on the use of anticoagulants at the baseline visit [13].
- Patients with symptoms (Sy) = 2: Treatment was based on a rhythm-control strategy during follow-up. Patients who switched from a rate-control strategy to rhythm control and those who maintained the baseline rhythm-control strategy during follow-up were considered as receiving rhythm-control treatment.
- Patients with substrate (Su) ≥ 1: The analysis was based solely on cardiovascular risk factors, and LA dilation was not considered. The cardiovascular risk factors considered were hypertension, diabetes mellitus, coronary artery disease, and heart failure (HF). Patients were considered to have risk factors treated based on the treatments received as follows:
  - Hypertension: Treatment with angiotensin-converting enzyme inhibitors/angiotensin II receptor blockers, non-dihydropyridine calcium channel blockers, or beta-blockers at the baseline visit.
  - Diabetes mellitus: Treatment with oral antidiabetic agents or insulin at baseline visit.
  - Coronary artery disease: Treatment with beta-blockers and statins at baseline visit.
  - HF: Treatment with ACE inhibitors/ARBs and beta-blockers at baseline visit.

Treatment of all risk factors was considered adequate in cases of patients whose risk factors were all well treated. Treatment of any risk factor was considered inadequate in cases where any risk factor was poorly treated

### 2.4. Statistical Analysis

Continuous variables are described as the mean ± standard deviation (SD) if normally distributed and the median and interquartile range. A *t* test was used for comparisons between groups. Categorical variables were expressed as frequencies and percentages, and the χ2 test or Fisher’s exact test was applied for comparisons between groups.

The results were expressed with *p*-values, 95% confidence intervals (CIs), and hazard ratios (HRs). Statistical significance was defined by a *p*-value < 0.05. Kaplan–Meier curves were plotted, and survival distributions were compared using the log-rank test. Pairwise multiple comparison techniques were applied between the different types of AF and adjusted using Bonferroni correction. Single-factor and multivariate Cox proportional hazards analyses were used to identify the prognostic value of the modified 4S-AF domains in terms of mortality, cardiovascular mortality, and thromboembolic events. For bleeding events, we used a Poisson regression model to take into account recurrent events.

## 3. Results

The analysis included 997 patients from the 1001 patients initially recruited in the REGUEIFA registry. Four patients were lost during follow-up. The baseline characteristics are shown in Table 1. The mean age was 67.6 ± 11.9 years, and one-third of the patients were women. One-third of the patients were recruited from hospitalization, and two-thirds were recruited from outpatient consultations. The most frequent risk factor was hypertension (62% of patients), and 15% had HF. Out of the patients with HF, 60% had a depressed left ventricular ejection fraction (LVEF). Furthermore, 6% of patients had a prior thromboembolic event, and 3.5% had a prior bleeding event. A rhythm-control strategy was established in 63.4% of the patients, and cardioversion was performed in 44% of the patients.

Nearly 41% of the patients were obese. LA was enlarged with a mean volume of 56 mL/m^2^ and a median volume of 46 mL/m^2^. Table 2 shows the distribution of cardiovascular risk factors and LA enlargement. It is noteworthy that only 17% had no cardiovascular risk factors, and 14% had a normal-sized atrium. However, more than 50% exhibited severe LA enlargement.

Two-thirds of patients were symptomatic with an EHRA score ≥ 2. The CHA_2_DS_2_-VASc score was 2.4 ± 1.5, and the HAS-BLED score was 0.7 ± 0.8. At baseline, 91% of the patients were taking oral anticoagulation medications, and 39% were taking antiarrhythmic drugs. Regarding the AF type, 22.7% of the patients had a first AF diagnosis, 22.6% had paroxysmal AF, 26.3% had persistent AF, 2.6% had long-lasting persistent AF, and 25.9% had permanent AF. There were 76 deaths (7.6% mortality rate), and nearly half stemmed from cardiovascular causes. There were 26 patients (2.6%) who had a thromboembolic event during follow-up. There were 81 bleedings in 71 patients during follow-up, and 27 (33.3%) were considered major bleedings (Table 2).

**Figure 1:**
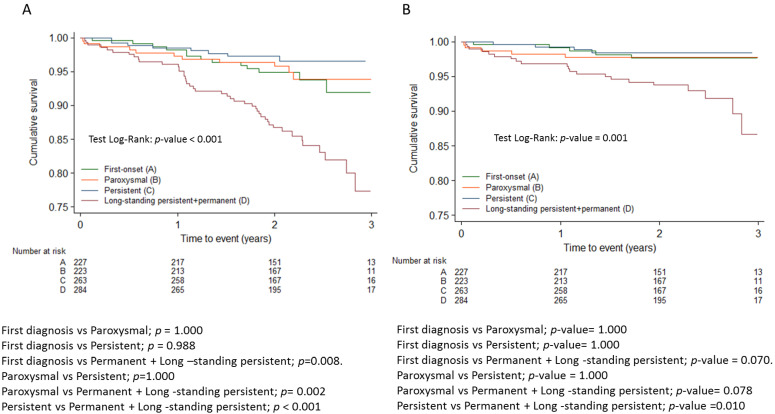
Kaplan-Meier survival curves for all cause mortality (**A**) and cardiovascular mortality (**B**) adjusted with Bonferroni correction.

**Figure 2:**
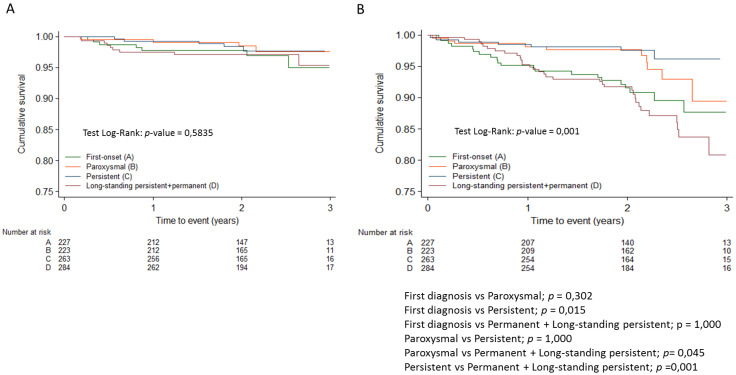
Kaplan-Meier survival curves for thromboembolic events (**A**) and hemorrhagic events (**B**) adjusted with Bonferroni correction.

Table 3 shows the distribution of registry patients according to the modified domains of the 4S scheme. Regarding the type of AF, the permanent form was associated with higher overall mortality and cardiovascular mortality than other forms of AF, including persistent AF (Figure 1). No differences in thromboembolic risk were detected between the different forms of AF, and both first-diagnosed and permanent forms were associated with higher hemorrhagic risk compared to the paroxysmal and persistent forms (Figure 2). None of the 29 patients with a score of 0 (no CV risk factors nor LA dilation) in the Su domain experienced any events. Due to the absence of events in this category, the comparison in the Su domain was made between patients with a score of 2 and those with a score of 1.

### 3.1. Modified 4S-AF Scheme and Outcomes

A multivariate Cox proportional hazards analysis was conducted to determine whether the domains of the modified 4S-AF scheme are independent predictors of all-cause mortality, cardiovascular mortality, and any thromboembolic event. The standard Cox proportional hazards models do not account for the possibility of a phenomenon of interest being recurrent and exclude events following the first occurrence and the times between occurrences. For this reason, a Poisson regression model was used to determine whether each of the 4S domains is independently associated with hemorrhagic events.

A multivariate Cox proportional hazards analysis was performed with all domains of 4S-AF considered as continuous variables. According to the results, all of the domains were independent predictors of all-cause mortality (St (HR 2.94, 95% CI: 1.64–5.29, Sy (HR 1.82, 95% CI: 1.40–2.37), Sb (HR 1.64, 95% CI: 1.26–2.14), and Su (HR: 1.27, 95% CI: 1.02–1.58)).

Table 4 summarizes the impact of the domains of the modified 4S-AF scheme on all-cause mortality. Multivariate Cox proportional hazards analysis was also performed with all domains of 4S-AF considered as categorical variables, including category 2 (non-anticoagulated patients with CHA_2_DS_2_-VASc > 1) in the stroke risk domain. The risk stratification for all-cause mortality improved by more than 2 times (St risk 2 vs.1: HR: 2.17; 95% CI; 1.03–4.55), *p =* 0.041). Patients with moderate and severe symptoms had a more unfavorable prognosis compared to asymptomatic patients (HR: 2.2; 95% CI; 1.3–3.9; *p* = 0.005 and HR: 3.4; 95% CI: 2.0–6.0; *p* < 0.001, respectively). In relation to the severity of AF, the mortality rate of patients with permanent AF was more than 2 times higher compared to patients with paroxysmal AF or a first episode of AF (HR: 2.5; 95% CI:1.5–4.2). However, no differences were detected between paroxysmal and persistent AF.

A multivariate Cox proportional hazards analysis was performed with all domains of 4S-AF considered as continuous variables. According to the results, symptoms and severity of burden and substrate were independent predictors of cardiovascular mortality (Sy (HR 2.3, 95% CI: 1.6–3.4, *p* < 0.001; Sb (HR 1.7, 95% CI: 1.1–2.5), *p* = 0.010; Su (HR 1.9, 95% CI: 1.3–2.7), *p* < 0.001).

Table 4 summarizes the impact of the domains comprising the modified 4S-AF scheme on cardiovascular mortality. Multivariate Cox proportional hazards analysis was also performed with all domains of 4S-AF considered as categorical variables. Patients with severe symptoms related to AF had a 6.5 times higher risk of cardiovascular mortality than asymptomatic patients, and those with permanent AF had almost a 3 times higher risk than patients with paroxysmal or first-episode AF.

The multivariate Cox proportional hazards analysis showed that none of the domains, considered continuous variables, were independent risk factors for thromboembolism.

According to the Poisson regression model, the stroke risk domain, considered as a continuous variable, was an independent factor of hemorrhagic events (HR: 2.8; 95% CI 1.7–4.7, *p <* 0.001). Table 4 summarizes the impact of the domains, considered as categorical variables, on hemorrhagic events as well. Notably, patients with St scores of 2 had a 29 times higher risk of hemorrhage compared to patients with scores of 0 (who had no anticoagulation at baseline) and a similar risk to patients with a score of 1 (baseline anticoagulation). Among the 40 patients with St scores of 2, 6 patients experienced hemorrhagic events during the 2-year follow-up. Of these, at baseline, two patients had antiplatelet therapy with aspirin, and one patient had dual antiplatelet therapy (aspirin + clopidogrel). Furthermore, two of these patients did not receive anticoagulation at baseline, but they did receive it during follow-up with vitamin K antagonist and dabigatran, respectively. The hemorrhagic events occurred after the initiation of anticoagulation in these two patients. Only one patient experienced a hemorrhagic event without undergoing any antithrombotic treatment.

Within the Sb domain, different categories exhibited distinct hemorrhagic risks. Specifically, patients with an Sb score of 2 (permanent AF) showed a 3 times higher risk than the persistent AF group (HR: 3.1; 95% CI 1.5–6.5, *p* = 0.002). Furthermore, their risk was 70% higher than that of the paroxysmal AF + first-episode AF group (HR:1.7; 95% CI 1.03–2.72, *p* = 0.030).

### 3.2. Effect of Treatment and 4S-AF

The impact of treatment of the 4S domains on all-cause mortality is summarized in Table 5. The multivariate Cox proportional hazards analysis was adjusted for age, gender, creatinine, chronic obstructive pulmonary disease, coronary heart disease, diabetes mellitus, heart failure, hypertension, peripheral vascular disease, previous thromboembolism, and sleep apnea. The results showed that the treatment with anticoagulation therapy for reducing stroke risk was the only independent domain treatment associated with a reduction in all-cause mortality (HR: 0.41; 95% CI 0.19–0.89 *p =* 0.023).

## 4. Discussion

The first main finding of our study is that all 4S-AF domains were independent factors of all-cause mortality when analyzed as continuous variables. The modification introduced in the stroke domain not only allowed for better AF characterization but may also help improve the risk stratification for mortality. Patients with CHA_2_DS_2_-VASc ≥ 1 and no oral anticoagulation showed a higher risk of death, as evidenced by HRs of 19 compared to the CHA_2_DS_2_-VASc = 0 and 2.2 compared to those with CHA_2_DS_2_-VASc ≥ 1 with oral anticoagulation. Symptomatic patients with EHRA scores of IIb or higher had higher mortality than asymptomatic patients and those with EHRA scores of IIa. Those with permanent AF had a 2.5 times higher risk of mortality compared to those with paroxysmal AF or first-episode AF.

Another main finding is that the Sy and Sb domains were independent factors of cardiovascular mortality. It is noteworthy that the permanent AF group’s HR was 2.8 times higher than that of the paroxysmal + first-episode AF group, but no differences were detected between the paroxysmal and persistent AF groups. The modified stroke risk was an independent factor for bleeding. Stroke risk was the only domain that was an independent factor for the treatment effect on overall mortality, and there was a 59% reduction in mortality associated with anticoagulation.

The 4S-AF score is for characterizing AF rather than predicting events. However, the incorporation of different domains, including comorbidities, systemic diseases, and biomarkers beyond the classic assessment of stroke and bleeding, makes it attractive for adding prognostic value [13,14,15,16] and predicting the progression of AF [17]. The improvement of the stroke risk domain by including an easily measurable parameter, such as anticoagulant treatment, allows for enhancement of the prognostic value of this domain. Thus, when we analyzed the effect of treatment on overall mortality, anticoagulant treatment was the only treatment domain that reduced mortality.

None of the treatments associated with the other domains achieved this. This result differs from that obtained in the EORP-AF study, where both anticoagulant treatment and rhythm control for patients with EHRA scores of III or IV were associated with prognostic improvement [13]. These differences may be explained by the low number of highly symptomatic patients (Sy = 2) in our series (*n* = 99), the high mortality in this group (20 patients, 25%), and the definition of rhythm-control strategy, which includes non-sinus rhythm.

The 2024 European guidelines have established AF-CARE as the framework for AF characterization [18]. They emphasize that comorbidities should be central to patient management, and their early and dynamic treatment is highly effective in preventing recurrences. However, treatment of the substrate in the REGUEIFA registry did not influence survival in contrast to anticoagulant therapy, which did. This may be explained by the fact that not all cardiovascular risk factors have the same impact on prognosis, the evaluation of a treatment considered effective, or the fact that anticoagulation remains the intervention with the greatest prognostic impact in AF patients.

In the comparison of REGUEIFA characteristics with other registries such as GARFIELD [19] and EORP-AF [13], REGUEIFA patients were younger and had lower rates of heart failure, coronary artery disease, and chronic kidney disease, as well as lower CHA_2_DS_2_-VASc and HAS-BLED scores (Table 6). The incidence of permanent AF was lower compared to EORP-AF but higher than in GARFIELD, which primarily included patients with a first episode of AF. The proportion of patients receiving anticoagulant and antiarrhythmic treatment was higher in REGUEIFA, likely due to the inclusion of 100% of patients from Cardiology Units.

The GARFIELD study assessed the incidence of stroke, death, or major bleeding over 2 years in a cohort of newly diagnosed AF patients. In that study, the rates of overall mortality (3.8/100 person-years) and cardiovascular mortality (1.5/100 person-years) were similar to those in the REGUEIFA registry (7.6% and 3.5% at two years, respectively) [19]. In the REGUEIFA study, the rate of stroke was nearly half that of GARFIELD (1.2/100 person-years vs. 1.3% at 2 years), which is probably due to the higher rates of anticoagulation in the REGUEIFA study (90% vs. 61%) and the higher rate of direct oral anticoagulant use (40% vs. 10%).

The rate of major bleeding in the GARFIELD study (0.70/100 person-years) was one-fourth of that in the REGUEIFA study (3% at 2 years) [19]. This effect is likely due to the higher rate of anticoagulation among REGUEIFA patients. Finally, oral anticoagulation in the GARFIELD study reduced overall mortality by 35%, while in the REGUEIFA study, it was reduced by 59%.

In the non-paroxysmal group, the risk of death was higher (HR: 1.45) in the GARFIELD study [19]. In the ENGAGE AF-TIMI 48 study, all-cause mortality was lower in paroxysmal cases (3.0%/year) compared with persistent cases (4.4%/year) and permanent AF (4.4%/year) [20]. In the ARISTOTLE trial, the rate of stroke or systemic embolism was significantly higher in patients with persistent or permanent AF than in patients with paroxysmal AF (1.5 vs. 0.98%). There was also a trend towards higher mortality in patients with persistent or permanent AF (3.9 vs. 2.8%) [21]. In the REGUEIFA registry, patients with permanent AF had a significantly higher risk of mortality and cardiovascular mortality than patients with paroxysmal AF or first AF episodes (HR: 2.5 and 2.8, respectively). Patients with permanent AF also exhibited significantly higher hemorrhagic risk than those with persistent AF (HR: 3.14) and paroxysmal, first-episode, and persistent AF (HR: 1.70).

We did not find differences between the paroxysmal and persistent forms as seen in the ENGAGE [20] or ARISTOTLE [21] studies, but there was clearly a worse prognosis in those with the permanent form. This suggests that the burden of AF is a primary determinant of prognosis, as demonstrated in a subset of the CASTLE AF study in patients with AF and HF with depressed LVEF [22]; in a more recent study (The Japanese Registry of Acute Decompensated Heart Failure), hospitalized patients with heart failure who had permanent AF exhibited an increased risk of cardiovascular mortality and heart failure readmission compared to patients without AF. This increased risk was not observed in patients with paroxysmal AF [23].

Patients with St scores of 2 had a higher risk of bleeding than non-anticoagulated patients and a similar risk to anticoagulated patients. Most of these patients with St scores of 2 and bleeding were undergoing antiplatelet therapy or dual antiplatelet therapy. It is well known that the risk of bleeding in patients on dual antiplatelet therapy (aspirin and clopidogrel) is similar to that of anticoagulated patients with vitamin K antagonists, as seen in the ACTIVE W trial. For patients with CHADS_2_ = 1, the annual major bleeding rate was 2.09% in the clopidogrel + aspirin group, exceeding the 1.36% observed in the oral anticoagulation group. Moreover, the absolute risk of major bleeding on oral anticoagulation was significantly lower in patients with CHADS_2_ = 1 compared to those with CHADS_2_ > 1, a difference that was not observed in the clopidogrel + aspirin group. [24] The bleeding risk associated with dual antiplatelet therapy is even higher compared to direct oral anticoagulants, as demonstrated in the recent EPIC-CAD study. This study, conducted in patients with atrial fibrillation at high thromboembolic risk and stable coronary artery disease, showed that dual antiplatelet therapy resulted in a similar rate of ischemic events but a threefold higher rate of bleeding events compared to edoxaban [25]. Additionally, patients on single antiplatelet therapy have a similar risk to patients on direct oral anticoagulants, as shown in the AVERROES trial [26].

### Limitations

The results of the REGUEIFA study may be influenced by a potential center-dependent effect as the volume of included patients varied among different recruiting centers. Although the consecutiveness of the included patients was pursued among recruiters, it could not be fully guaranteed. Patients with HF and preserved left ventricle systolic function accounted for only 40% of overall HF patients. This figure may seem low and is attributed to many of these patients being treated outside of cardiology clinics. The number of women was also lower than desired.

## 5. Conclusions

Oral anticoagulation for stroke risk management was the only 4S-AF treatment domain associated with a reduction in mortality. The modification of the stroke risk score with a category reflecting anticoagulation status may help better characterize and stratify overall mortality risk, as shown in the contemporary AF cohort of the REGUEIFA study. The permanent form of AF was associated with a higher risk of overall mortality and cardiovascular mortality.

## Figures and Tables

**Table 1:** Baseline characteristics of the study population.

Baseline Characteristics, *n* (%)	(*n* = 997)
Age (y)	
Mean ± SD	67.6 ± 11.9
Median (IQR)	68 (59–77)
Sex. female	321 (32.2)
Site of inclusion	
Hospitalized	341 (34.2)
Consultation	656 (65.8)
BMI (kg/m^2^)	29.6 ± 5.0
Hypertiroidism	19 (1.9)
Hypotiroidism	65 (6.5)
Dementia	7 (0.7)
Anemia	40 (4.0)
Valvular heart disease	114 (11.4)
Cardiomyopathy	78 (7.8)
Prior thromboembolic events	61 (6.1)
Ischemic stroke	29 (2.9)
Peripheral embolism	5 (0.5)
TIA	18 (1.8)
Pulmonary embolism/deep venous thrombosis	14 (1.4)
Prior bleeding	35 (3.5)
Type of bleeding	
Major	15 (42.9)
Relevant non-major	20 (57.1)
CHA_2_DS_2_-VASc	2.4 ± 1.5
HASBLED	0.7 ± 0.8
Prior electrical cardioversion	225/620 * (36.3)
Prior cardioversion	278/620 * (44.8)
Prior PVI ablation	76/620 * (12.2)
Creatinine, mg/dL	0.9 ± 0.3
LA diameter, mm ± SD	44.1 ± 11.6
LA volume, mean (mL/m^2^) ± SD	56.7 ± 32.9
LVEF,% ± SD	55.9 ± 12.7
**4S Substrate (Su)**	
*Risk factor*	
Obesity (BMI ≥ 30)	408 (40.9%)
HF	148 (14.8)
HFpLVEF >50%	59 (39.8)
HFrLVEF: <40%	56 (37.8)
HFmLVEF: 40–50%	29 (19.6)
Diabetes	189 (18.9%)
Coronary heart disease	115 (11.5%)
Peripheral vascular disease	29 (2.9%)
Previous thromboembolic events	61 (6.1%)
Chronic kidney disease	64 (6.4%)
Neoplasia	83 (8.3%)
Hypertension	622 (62.4%)
COPD or OSA	145 (14.5%)
*CV risk factors*	
No risk factors	171 (17.1)
1 risk factor	273 (27.4)
Risk factors (≥2)	553 (55.5)
*LA dilation*	
No dilation	141 (14.1)
Mild/moderate LA dilation	281(28.2)
Severe LA dilation	575 (57.7)
**4S Symptoms (Sy)**	
EHRA classification	
I	336 (33.7)
II a	389 (39.0)
II b	170 (17.1)
III	95 (9.5)
IV	7 (0.7)
**4S Severity of Burden**	
First episode	227 (22.7)
Paroxysmal	223 (22.6)
Persistent	263 (26.3)
Permanent or long-lasting	284 (28.4)
**Rhythm interventions**	545 (54.7)
Rate control	365 (36.6)
Rhythm control	632 (63.4)
ECV	283 (28.3)
Pharmacological CV	26 (2.6)
PVI	167 (16.7)
AV node ablation	9 (0.9)
Beta-blockers	680 (68.2)
Calcium antagonists	54 (5.4)
Digoxin	61 (6.1)
Antiarrhythmic drugs	387 (38.8)
Amiodarone	182 (47.0)
Flecainide	180 (46.5)
Propafenone	5 (1.3)
Dronedarone	6 (1.5)
Sotalol	13 (3.4)
Ranolazine	1 (0.3)
Anticoagulation therapy	898 (90.1)
Vitamin K antagonists	527 (58.7)
DOACS	371 (41.3)

BMI: body mass index; COPD: chronic obstructive pulmonary disease; OSA: obstructive sleep apnea; HF: heart failure; HFpLVEF: HF preserved left ventricular ejection fraction; HFrLVEF: HF reduced LVEF; HFmLVEF: HF mildly reduced LVEF; TIA: transient ischemic attack; EHRA: European Heart Rhythm Association; PVI: pulmonary vein ablation; AV: atrioventricular; LA: left atrium; ECV; electrical cardioversion; CV: cardioversion.* *n* = 997—patients with first-episode AF.

**Table 2:** Adverse events during follow-up.

Event	*n (%)*
All-cause mortality	76 (7.6)
CV mortality	35 (3.5)
TE events	26 (2.6)
Ischemic stroke	13 (1.3)
Peripheral embolism	5 (0.5)
TIA	6 (0.6)
PE/DVT	2 (0.2)
Hemorrhagic events, *n* *	81 (7.1)
Type of bleeding	
Major	27 (33.3)
Non-major	54 (66.7)
Relevant non-major	33 (44.4)
Intracranial hemorraghes	4 (4.9)
Major extracranial hemorraghes	23 (28.4)
Major extracranial localization	
Gastrointestinal superior	4 (17.4)
Gastrointestinal inferior	8 (34.8)
Soft tissue, muscle, and skin	5 (21.7)
Nasal	3 (13.0)
Splenic	1 (4.4)
Other	2 (8.7)

CV: cardiovascular; TE: thromboembolic; TIA: transient ischemic attack; PE/DVT: pulmonary embolism/deep venous thrombosis. * 81 hemorrhagic events in 71 patients. % expressed over the total study population.

**Table 3:** Definition of 4-S classification scheme and patient distribution according to 4S-AF scheme.

Domain	Sub-Domain	Scoring	Interpretation	Definition	*n* (%)
**Stroke risk (St)**		0	Low risk	CHA_2_DS_2_-VASc = 0 in men or CHA_2_DS_2_-VASc = 1 in women	177 (17.7)
		1	No low risk	CHA_2_DS_2_-VASc ≥ 1 in men or CHA_2_DS_2_-VASc ≥ 2 in women and under baseline anticoagulant treatment	774 (77.6)
		2	High risk	CHA_2_DS_2_-VASc ≥ 1 in men or CHA_2_DS_2_-VASc ≥ 2 in women without baseline anticoagulant treatment	46 (4.7)
**Symptoms (Sy)**		0	No or mild symptoms	EHRA I–IIa	725 (72.7)
		1	Moderate symptoms	EHRA IIb	170 (17.1)
		2	Severe or debilitating symptoms	EHRA III–IV	102 (10.2)
**Severity of AF burden (Sb)**		0	New AF episodes, short or infrequent episodes	First diagnosis of AF or paroxysmal	450 (45.1)
		1	Intermediate episodes or frequent episodes of AF	Persistent AF	263 (26.4)
		2	Long AF episodes or very frequent episodes	Long-lasting AF or permanent AF	284 (28.5)
**Substrate (Su)**		0	No CV risk factors nor LA dilation		29 (2.9)
		1	1 CV risk factor or mild or moderate LA dilation		96 (9.7)
		2	1 CV risk factor and mild or moderate LA dilation or multiple CV risk factors or severe LA dilation		211 (21.2)
		3	1 CV risk factor and severe LA dilation or multiple CV risk factors and mild/moderate LA dilation		352 (35.3)
		4	Multiple CV risk factors and severe LA dilation		309 (30.9)

CV: cardiovascular. LA: Lef atrium. LA dilation: No LA dilation: LA diameter < 40 mm or LA volume ≤ 35 mL/m^2^. Mild/moderate LA dilation: LA diameter 40–49 mm or LA volume: 36–48 mL/m^2^. Severe LA dilation: LA diameter ≥ 50 mm or LA volume 48 > mL/m^2^.

**Table 4:** Univariate and multivariate Cox regression analysis of the 4S domains scheme for all-cause mortality, cardiovascular mortality, thromboembolic events, and hemorrhagic events.

	Univariate Analysis		Multivariate Analysis ^a^	
	All-Cause Death			
	HR (95% CI)	*p*-Value	HR (95% CI)	*p*-Value
Stroke risk (St)				
1 vs. 0	14.9 (2.1–107.3)	0.007	8.8 (1.2–64.9)	0.032
2 vs. 0	31.2 (3.9–249.2)	0.001	19.1 (2.4–154.7)	0.006
Symptoms (Sy)				
1 vs. 0	2.2 (1.3–3.9)	0.005	2.2 (1.3–3.9)	0.005
2 vs. 0	4.0 (2.3–6.8)	<0.001	3.4 (2.0–6.0)	<0.001
Severity of AF burden (Sb)				
1 vs. 0	0.6 (0.3–1.3)	0.182	0.6 (0.3–1.4)	0.270
2 vs. 0	2.9 (1.7–4.7)	<0.001	2.5 (1.5–4.2)	<0.001
Substrate (Su)				
2 vs. 1	1.7 (1.0–2.9)	0.065	1.3 (0.8–2.2)	0.350
	**Cardiovascular mortality**			
	HR (95% IC)	*p*-Value	HR (95% IC)	*p*-Value
Stroke risk (St)				
1 vs. 0	7.0 (1.0–51.0)	0.056	3.0 (0.4–23.1)	0.289
2 vs. 0	11.9 (1.2–114.8)	0.032	5.3 (0.5–52.4)	0.154
Symptoms (Sy)				
1 vs. 0	3.0 (1.3–7.0)	0.011	3.0 (1.3–7.0)	0.012
2 vs. 0	7.3 (3.4–15.8)	<0.001	6.5 (3.0–14.1)	<0.001
Severity of AF burden (Sb)				
1 vs. 0	0.7 (0.2–2.2)	0.520	0.7 (0.2–2.4)	0.613
2 vs. 0	3.3 (1.5–7.0)	0.002	2.8 (1.3–6.0)	0.008
Substrate (Su)				
2 vs. 1	2.8 (1.1–7.3)	0.032	2.2 (0.8–5.6)	0.111
	**Thromboembolic event**			
	HR (95% IC)	*p*-Value	HR (95% IC)	*p*-Value
Stroke risk (St)				
1 vs. 0	2.4 (0.6–10.1)	0.242	2.1 (0.5–9.6)	0.330
2 vs. 0	3.8 (0.5–26.9)	0.184	3.5 (0.5–25.3)	0.221
Symptoms (Sy)				
1 vs. 0	0.2 (0.03–1.6)	0.136	0.2 (0.03–1.6)	0.129
2 vs. 0	1.9 (0.7–4.9)	0.217	1.7 (0.6–4.5)	0.296
Severity of AF burden (Sb)				
1 vs. 0	0.8 (0.3–2.2)	0.647	0.8 (0.3–2.4)	0.700
2 vs. 0	1.4 (0.6–3.3)	0.443	1.3 (0.5–3.1)	0.580
Substrate (Su)				
2 vs. 1	1.1 (0.5–2.4)	0.888	0.9 (0.4–2.2)	0.870
	**Hemorrhagic event**			
	IRR (95% IC)	*p*-Value	IRR (95% IC)	*p*-Value
Stroke risk (St)				
1 vs. 0	16.4 (2.3–118.0)	0.005	18.8 (2.6–137.3)	0.004
2 vs. 0	27.9 (3.4–226.5)	0.002	29.2 (3.6–239.7)	0.002
Symptoms (Sy)				
1 vs. 0	1.4 (0.8–2.5)	0.188	1.5 (0.8–2.5)	0.170
2 vs. 0	1.9 (1.0–3.5)	0.038	1.6 (0.9–3.0)	0.110
Severity of AF burden (Sb)				
1 vs. 0	0.4 (0.2–0.9)	0.029	0.5 (0.3–1.1)	0.103
2 vs. 0	1.7 (1.1–2.7)	0.028	1.7 (1.0–2.7)	0.030
Substrate (Su)				
2 vs. 1	0.8 (0.5–1.3)	0.357	0.7 (0.4–1.0)	0.075

AF: atrial fibrillation; HR: hazard ratio; CI: confidence interval; IRR: incidence rate ratio, ^a^ Adjusted for each domain within the 4S-AF scheme. -St: score of 0: CHA_2_DS_2_-VASc = 0 in men or CHA_2_DS_2_-VASc = 1 in women; score of 1: CHA_2_DS_2_-VASc ≥ 1 in men or CHA_2_DS_2_-VASc ≥ 2 in women and under baseline anticoagulant treatment; score of 2: CHA_2_DS_2_-VASc ≥ 1 in men or CHA_2_DS_2_-VASc ≥ 2 in women without baseline anticoagulant treatment. Sy: score of 0: EHRA I or IIa; score of 1: EHRA IIb; score of 2: EHRA III or IV. Sb: score of 0: first AF diagnosis or paroxysmal AF; score of 1: persistent AF; score of 2: long-lasting persistent AF or permanent AF. Su: score of 0: patients with 0 points; score of 1: patients with 1 or 2 points; score of 2: patients with 3 or 4 points. Due to the absence of events in patients with a score of 0 in the substrate domain, the comparison is made between patients with a score of 2 and score of 1.

**Table 5:** Effect of the treatment according to 4S scheme on all-cause mortality.

Treatment	HR ^a^ (95% CI)	*p*-Value
St ≥ 1, Anticoagulant therapy (*n* = 778; *n* events = 74)	0.41 (0.19–0.89)	0.023
Sy = 2, Implementation of a rhythm-control strategy during follow-up (*n* = 99; *n* events = 20)	1.01 (0.28–3.66)	0.988
Su ≥ 1 *; CV risk factors treatment (*n* = 672; *n* events = 65)	0.90 (0.50–1.60)	0.719

HR: hazard ratio; CI: confidence interval; CV: cardiovascular. ^a^ Adjusted for age, gender, creatinine, chronic obstructive pulmonary disease, coronary heart disease, diabetes mellitus, heart failure, hypertension, peripheral vascular disease, previous thromboembolism, and sleep apnea. * Based on CV risk factors only.

**Table 6:** Baseline characteristics of EORP-AF, GARFIELD, and REGUEIFA.

	REGUEIFA	EORP-AF	GARFIELD
Size, pt	1.001	11.096	17.162
Age, y	67.6 (11.9)	71 (63–77)	69.8 (11.4)
Sex female, %	32.2	40.7	43.8
CHA_2_DS_2_VASc	2.4 (1.5)	3.1 (1.7)	3.3 (1.6)
HASBLED	0.7 (0.8)	1.5 (1.0)	1.5 (0.9)
Congestive heart failure, (%)	14.8	39.5	20.6
Coronary artery disease, (%)	11.5	29.3	19.9
HTN, (%)	62.4	62.1	78.1
Stroke/TIA, (%)	4.7	9.2	12.6
History of bleeding (%)	3.5	5.2	2.9
CKD (%)	6.4	12.5	10.3
Type of AF			
Paroxysmal, (%)	22.6	25.7	25.2
Persistent, (%)	26.3	19.1	15.6
Permanent, (%)	28.4	37.8	13.1
First AF, (%)	22.7	15.6	46.1
OAC therapy	90.1	84.9	60.8
Antiarrhythmic drugs therapy	38.8	27.8	na
Care setting specialty Cardiologist, (%)	100	68.5	61.9

AF: atrial fibrillation; HTN: hypertension; OAC: oral anticoagulation.

## Data Availability

The data that support the findings of this study are available on request from the corresponding author.
